# A new meningococcal B vaccine

**DOI:** 10.1186/1824-7288-40-S1-A2

**Published:** 2014-08-11

**Authors:** Rocco Russo

**Affiliations:** 1 Maternity and Pediatrics Services, Local Health Units Benevento, Italy; 2 Maternity and Pediatrics Services – Local Health Units Naples1, Italy

## 

Neisseria meningitidis is an important cause of invasive bacterial infection in children worldwide, and is a rare example of a bacterium that has evolved to become an obligate human commensal which commonly colonizes the oropharyngeal mucosa. Carriage is age-dependent and appears to be very common in young adults. The relationships between carriage and invasive disease are not completely understood [1].

Each year approximately 1.2 million cases of invasive meningococcal disease (sepsis or meningitis) with 135,000 deaths are estimated to occur worldwide [2].

**Figure 1:**
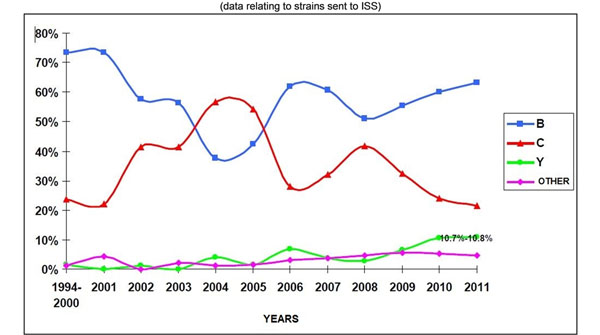
Variations in the proportion of strains of serogroup B, C and Y circulating in Italy

Epidemiology and serogroup distribution differs geographically, as the example in Figure 1 shows, with invasive disease mainly affecting young children, older children and young adults. In addition to age, another individual risk factor includes underlying immune deficiencies; the deficiency of complement components is a known risk factor for invasive infection. Crowding and concurrent upper respiratory tract infections might also contribute to the disease.

In early 2013, a new vaccine developed specifically to prevent disease caused by group B meningococci (MenB) was licensed in Europe (4CMenB, Bexsero^©^, Novartis Vaccines, Italy). This vaccine is protein-based and, therefore, compared to meningococcal conjugate vaccines , has a different mechanism of action, along with different safety, reactogenicity and immunogenicity profiles in the various age groups.

The vaccine, developed by reverse vaccinology, contains three surface-exposed recombinant proteins (fHbp, NadA, and NHBA) and outer membrane vesicles derived from the NZ98/254 strain and has the potential to reduce mortality and morbidity associated with serogroup B meningococci infections, but uncertainty remains about the breadth of protection the vaccine might induce against the diverse serogroup B meningococci strains that cause disease: Meningococcal Antigen Typing System predicted that 78% of all MenB strains would be killed by postvaccination [3].

In Italy (updated April 2014) the Basilicata Region [4] recommend Bexsero for the routine vaccination of infants and will have an active call to parents that includes providing the vaccine free of charge. The Board of Calendario per la Vita, comprising of the country’s foremost scientific societies, has recommended Bexsero for all infants with three doses in the first year of life and one dose at 13 months of age [5].

For parents and clinicians, the predicted benefits of 4-component meningococcal group B vaccine (4CMenB) outweigh existing uncertainties about the potential impact of the MenB vaccine against invasive disease, but future introduction of the vaccine must be followed by rigorous post-implementation surveillance to assess its value to health systems.
